# 

**Published:** 1902-11

**Authors:** 


### BUFFALO MEDICAL JOURNAL

**Established 1845 by AUSTIN FLINT, M. D.**

**EDITOR:**

WILLIAM WARREN POTTER, M. D.

**ASSISTANT EDITORS:**

WILLIAM C. KRAUSS, M. D. NELSON W. WILSON, M. D.

**ASSOCIATE EDITORS:**

JAMES WRIGHT PUTNAM, M. D.
ERNEST WENDE, M. D.
JOHN PARMENTER, M. D.
JOHN A. MILLER, PH. D
HARVEY R. GAYLORD, M. D.
MAUD J. FRYE, M. D.

SCRIPTA FERUNT ANNOS

LIBRARY
SURGEON GENERAL'S O[illegible]

EUROPEAN AGENCY, J. B. BAILLIERE & FILS, 19 Rue Hautefeuille, Paris.

*Subscription, if paid in advance, $2.00; otherwise, $2.50. Foreign subscription, $2.50*

Entered at the Post Office at Buffalo, N. Y., as second-class mail matter.

A. T. BROWN PRINTING HOUSE, BUFFALO, N. Y.

**Table of Contents on Page V.**

